# Developing a strain‐dependent susceptibility to hepatocellular carcinoma in a murine model based on histopathological evidence and genetic context

**DOI:** 10.1002/ame2.70283

**Published:** 2026-09-24

**Authors:** Osayd Zohud, Aysar Nashef, Imad Abu‐Elnaaj, Fuad A. Iraqi

**Affiliations:** ^1^ Department of Clinical Microbiology and Immunology, Gray Faculty of Medicine and Health Sciences Tel‐Aviv University Tel‐Aviv Israel; ^2^ Department of Oral and Maxillofacial Surgery Baruch Padeh Medical Center Poriya Israel; ^3^ Azrieli Faculty of Medicine Bar‐Ilan University Ramat Gan Israel; ^4^ Department of Oral and Maxillofacial Surgery Meir Medical Center, Affiliated to the Gray Faculty of Medicine and Health Sciences, Tel‐Aviv University Kfar Saba Israel

**Keywords:** CC mice, genetic susceptibility, hepatocellular carcinoma, heterozygous *Smad4* deletion, liver cancer, mouse model, *Smad4*, systems genetics, TGF‐β signaling, tumor modifiers

## Abstract

Hepatocellular carcinoma is a leading cause of cancer‐related mortality worldwide. *Smad4*, a well‐established tumor suppressor within the transforming growth factor‐β (TGF‐β) signaling pathway, has an oncogenic potential of its heterozygous loss, still poorly defined, especially across genetically diverse backgrounds. The aim was to investigate whether heterozygous loss of *Smad4* increases susceptibility to spontaneous liver tumor development in a genetically diverse murine population. We generated 260 F1(C57BL/6J/*Smad4* ^+/−^ X CC) mice, including 131 females and 129 males, by crossing *Smad4* ^+/−^ males (C57BL/6J background) with females from 14 Collaborative Cross (CC) lines. Mice were maintained and monitored under standardized conditions for up to 80 weeks. Liver tissues were analyzed by gross pathology and histology for evidence of neoplasia. Tumor incidence was calculated per CC line; and Fisher's exact tests were used to evaluate line‐specific susceptibility. Liver tumors developed in *Smad4* ^+/−^ mice (8/260; 3.08%) background, with no tumors observed in wild‐type littermates. Four CC lines exhibited tumor‐positive, with elevated incidence in CC025 (15.79%, *p* = 0.0011), CC010 (8.33%, *p* = 0.0173), and CC005 (6.90%, *p* = 0.0241). After Bonferroni correction for multiple comparisons, only CC025 remained statistically significant. Histopathology confirmed hepatocellular carcinoma in representative cases. Tumor incidence showed no sex bias and was restricted to specific genotypic contexts, indicating an interaction between heterozygous *Smad4* deletion and host genetic background. Partial loss of *Smad4* promotes liver carcinogenesis in a strain‐dependent manner, highlighting the importance of genetic modifiers in tumor susceptibility. The use of CC mice provides a valuable platform for uncovering genotype‐specific cancer susceptibilities in preclinical research.

## Graphical abstract

Using F1 (C57BL/6J/*Smad4* ^+^/^−^ X CC) mice, we found that partial loss of *Smad4* promotes liver carcinogenesis in a strain‐dependent manner, highlighting the importance of genetic modifiers in tumor susceptibility. The use of Collaborative Cross mice provides a valuable platform for uncovering genotype‐specific cancer susceptibilities in preclinical research.

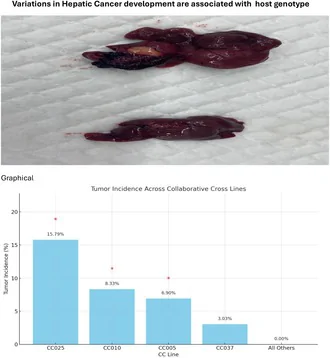

## 1 INTRODUCTION

Hepatocellular carcinoma (HCC) is the most common form of primary liver cancer and a major global health burden, accounting for over 800 000 deaths annually worldwide.^1^ The pathogenesis of HCC is driven by multifactorial processes involving chronic liver injury, inflammation, fibrosis, and cumulative genetic and epigenetic alterations.^2^ Among the central signaling pathways implicated in liver tumorigenesis is the transforming growth factor‐β (TGF‐β)/SMAD signaling cascade, which plays a dual role in liver biology, acting as a tumor suppressor in early stages and a promoter of tumor progression and metastasis in advanced disease.^3^, ^4^

*Smad4*, also known as *DPC4* (deleted in pancreatic carcinoma locus 4), is a critical mediator of canonical TGF‐β and BMP signaling pathways. It forms complexes with phosphorylated receptor‐regulated *Smad*s (R‐*Smad*s: *Smad2/3* or *Smad1/5/8*) that translocate into the nucleus and regulate the transcription of genes involved in cell cycle control, differentiation, apoptosis, and immune modulation.^5^, ^6^ Loss or mutation of *Smad4* disrupts this signaling balance and has been associated with increased susceptibility to various epithelial cancers, including pancreatic, colorectal, and gastric carcinomas.^7^ In the context of HCC, *Smad4* deficiency has been shown to cooperate with oncogenic insults (e.g., Ras activation, β‐catenin mutation) to promote hepatocarcinogenesis in murine models.^8^, ^9^

Despite its known tumor‐suppressive function, the impact of partial *Smad4* loss (heterozygosity) on liver cancer susceptibility remains poorly understood, particularly when modulated by host genetic background. Traditional mouse models, often limited by narrow genetic variation, fail to capture the diversity of tumor phenotypes seen in humans. To overcome this, the Collaborative Cross (CC) mouse resource was developed as a powerful panel of recombinant inbred strains derived from eight genetically diverse founder strains (A/J, C57BL/6J, 129S1/SvImJ, NOD/ShiLtJ, NZO/HILtJ, CAST/EiJ, PWK/PhJ, and WSB/EiJ) representing over 50 million segregating genetic variants.^10^, ^11^ CC mice have proven to be an exceptional tool for studying complex trait genetics, including host–pathogen interactions, metabolic diseases, immune response variability, and cancer susceptibility.^12^, ^13^

In our prior research, we investigated the phenotypic consequences of *Smad4* heterozygous knockout (*Smad4* ^+/−^) in first‐generation (F1) offspring derived from crosses between C57BL/6J‐*Smad4* ^+/−^ mice and multiple CC lines. This approach enabled the analysis of host genetic background influences on body weight trajectories, pancreatic development, and juvenile polyposis formation under controlled environmental conditions.^14^, ^15^, ^16^ Unexpectedly, during these experiments, we observed that specific CC lines exhibited a significantly increased incidence of spontaneous liver carcinoma, a phenotype not seen in other lines or in wild‐type (WT) littermates, under the same conditions and without any chemical induction.^17^, ^18^, ^19^

Unexpectedly, spontaneous liver tumors were observed in a subset of heterozygous *Smad4* knockout mice maintained on distinct CC‐derived genetic backgrounds. Therefore, the present study provides a descriptive analysis of strain‐dependent susceptibility to spontaneous hepatocellular carcinoma in genetically diverse *Smad4* ^+/−^ F1 mice. By integrating phenotypic observations and histopathological findings, we aimed to characterize this phenotype and to highlight the potential contribution of host genetic background to liver tumor susceptibility. Although several signaling pathways have been implicated in liver tumorigenesis and may interact with TGF‐β/SMAD signaling,^20^, ^21^, ^22^, ^23^, ^24^, ^25^ the present study was not designed to investigate these mechanisms directly. Thus, the findings presented here provide a framework for future studies aimed at identifying the genetic and molecular determinants underlying the observed strain‐dependent differences.

## 2 MATERIALS AND METHODS

### 2.1 Ethical approval and animal welfare

All procedures were approved by the Institutional Animal Care and Use Committee (IACUC) at Tel Aviv University under protocol no. 01‐19‐044. Experiments were conducted in compliance with the Guide for the Care and Use of Laboratory Animals (National Research Council, USA). Mice were monitored daily for general health and well‐being, with humane endpoints defined based on sustained weight loss, inactivity, or signs of distress. Animals meeting these criteria were euthanized using CO_2_ inhalation followed by cervical dislocation.

### 2.2 Mouse cohort and breeding strategy

A cohort of 499 F1 mice was generated, including 260 *Smad4* ^+/−^ mice (131 females and 129 males) and 239 *Smad4* ^+/+^ (129 females and 110 males), by crossing male C57BL/6J‐*Smad4* ^+/−^ mice (strain: B6.129‐*Smad4* ^tm1Mak^; Jackson Laboratory, Stock #029250) with female mice from 14 CC inbred lines. This breeding strategy produced genetically diverse F1 (*Smad4* ^+/−^ × CC) offspring, allowing analysis of host genetic background on tumor susceptibility. All F1 progeny were genotyped for the *Smad4* allele at weaning.

### 2.3 Genotyping of F1 mice

Tail biopsies were collected from all mice at 3–4 weeks of age. Genomic DNA was extracted using the NaOH method, and polymerase chain reaction (PCR) genotyping was performed using the following primers:

(1) Primer 30403 (WT specific): 5′‐TGT AGT TCT GTC TTT CCT TCC TG‐3′; (2) primer 30404 (common reverse): 5′‐ACT GAC CTT TAT ATA CGC GCT TG‐3′; and (3) primer oIMR2088 (KO specific): 5′‐AGA CTG CCT TGG GAA AAG CG‐3′.

Reactions were performed at a 25‐μL volume, as previously described.^14^, ^15^, ^16^ PCR products were analyzed using agarose gel electrophoresis. WT alleles produced a 200‐bp band, whereas KO alleles produced a 300‐bp band.^14^, ^15^, ^16^

### 2.4 Mouse housing conditions and monitoring

Mice were housed in sex‐separated, individually ventilated cages (IVC) with hardwood bedding under a 12‐h light/dark cycle at 22°C. Animals had ad libitum access to standard chow (TD.2018SC, Teklad Global), containing approximately 18.6% protein, 6.2% fat, and 44.2% carbohydrate by weight, and water. Body weight was recorded weekly until 16 weeks and then monthly until the study endpoint at 80 weeks. Any animals reaching predefined euthanasia criteria were humanely killed by CO_2_ inhalation.

### 2.5 Necropsy and liver assessment

At week 80, or earlier, if mice met predefined humane endpoints, all animals underwent full necropsy. Humane endpoints included signs of sustained weight loss (>20% baseline), severe lethargy, reduced mobility, ruffled fur, or visible distress, as assessed by daily monitoring. Mice meeting these criteria were euthanized humanely via CO_2_ inhalation followed by cervical dislocation. All animals underwent a complete necropsy. Liver morphology was assessed grossly, and any visible lesions, nodules, or abnormalities were recorded. Although histological confirmation of carcinoma was available only for a subset of mice, lesions exceeding 3 mm with irregular morphology and solid mass consistency were considered likely neoplastic based on previous criteria for spontaneous liver tumor classification in mice.^26^, ^27^, ^28^

### 2.6 Tumor scoring and incidence calculation

Liver lesions were evaluated by two independent investigators blinded to genotype and CC line identity. Each animal was scored as either “tumor‐positive” or “no tumor”.^26^, ^27^, ^28^

**FIGURE 1:**
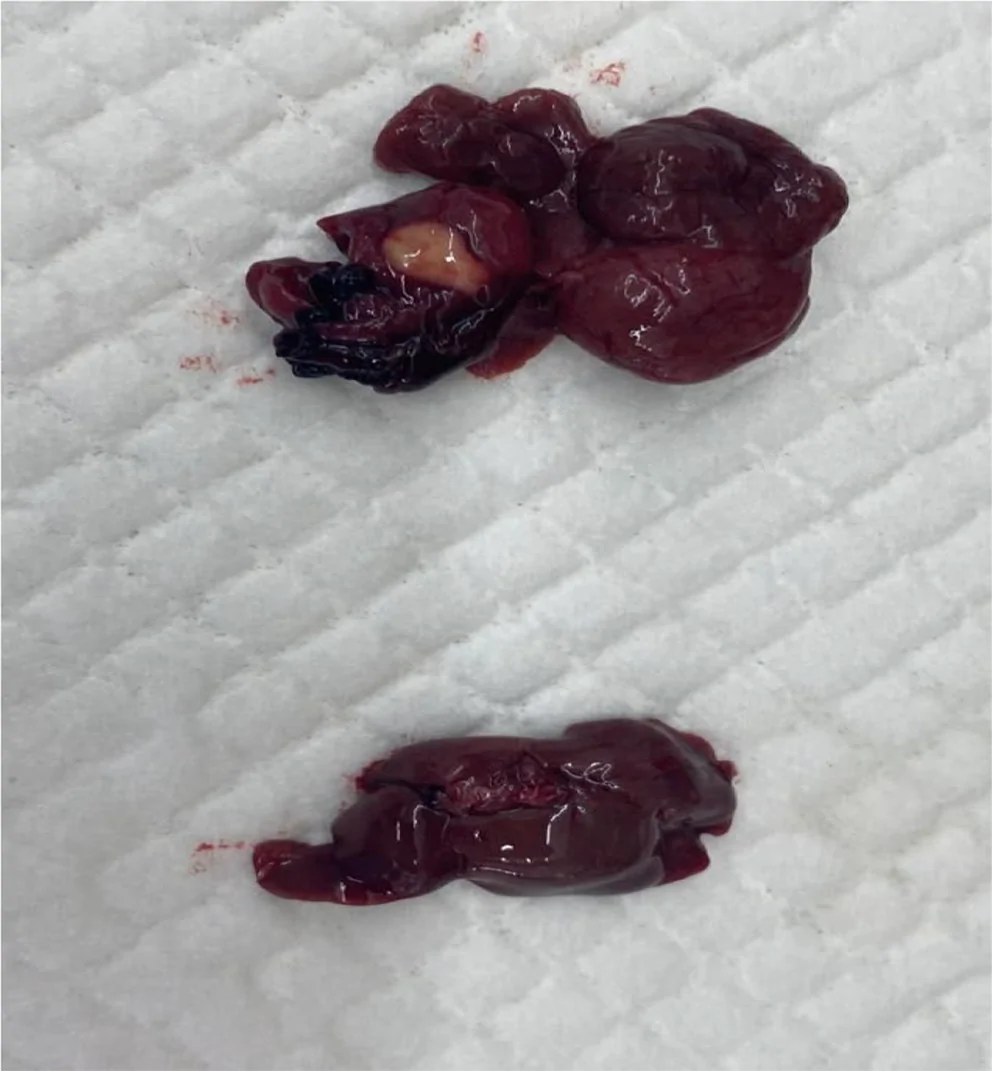
Gross morphology of a tumor‐bearing liver compared to a normal liver. The top specimen shows a liver from a *Smad4* ^+/−^ F1 mouse exhibiting multiple large, irregular, and discolored nodules consistent with hepatocellular carcinoma. The bottom specimen represents a normal liver from a wild‐type littermate, demonstrating a smooth surface and homogeneous red coloration.

A lesion was classified as tumor‐positive if it was ≥3 mm in diameter, exhibited irregular morphology or color, and had firm mass‐like consistency. Digital images were captured for each lesion and archived with size and location data. Figure 1 illustrates a representative tumor‐bearing liver (top) displaying multiple nodular masses and color heterogeneity in contrast to a smooth, uniformly pigmented control liver (bottom).^29^, ^30^, ^31^

To confirm neoplastic identity, a representative subset of grossly abnormal livers underwent histological evaluation. Lesions were fixed in 10% neutral‐buffered formalin for 24–48 h, embedded in paraffin, sectioned at 4 μm, and stained with hematoxylin and eosin (H&E) using standard protocols. Histological examination was conducted by a trained pathologist blinded to genotype and experimental group, using a Nikon Eclipse E600 microscope. Diagnostic features of HCC included trabecular or pseudoglandular architecture, increased nuclear‐to‐cytoplasmic ratio, cellular pleomorphism, and vascular invasion.^32^

Tumor incidence was calculated as the proportion of affected mice per CC line, stratified by *Smad4* genotype (WT vs. *Smad4* ^+/−^). Fisher's exact test was used to compare tumor incidence in each CC line against the pooled remainder of the *Smad4* ^+/−^ cohort. To account for multiple line‐specific comparisons across the 14 CC lines, Bonferroni correction was applied, resulting in an adjusted significance threshold of *p* < 0.00357 (0.05/14). Sex and age at necropsy were also recorded. Tumor size and gross morphological features were recorded digitally and archived.

## 3 RESULTS

To explore the influence of host genetic background on liver tumor susceptibility in the context of heterozygous *Smad4* deletion, we analyzed liver tissues from a cohort of 499 F1 mice generated by crossing *Smad4* ^+/−^ C57BL/6J males with females from 14 genetically diverse CC lines. Of these, 260 mice were heterozygous for *Smad4* (*Smad4* ^+/−^), and 239 were WT littermates. All mice were maintained under uniform housing and diet conditions and monitored longitudinally for up to 80 weeks. Complete necropsy was performed at the study endpoint or upon humane euthanasia.

**FIGURE 2:**
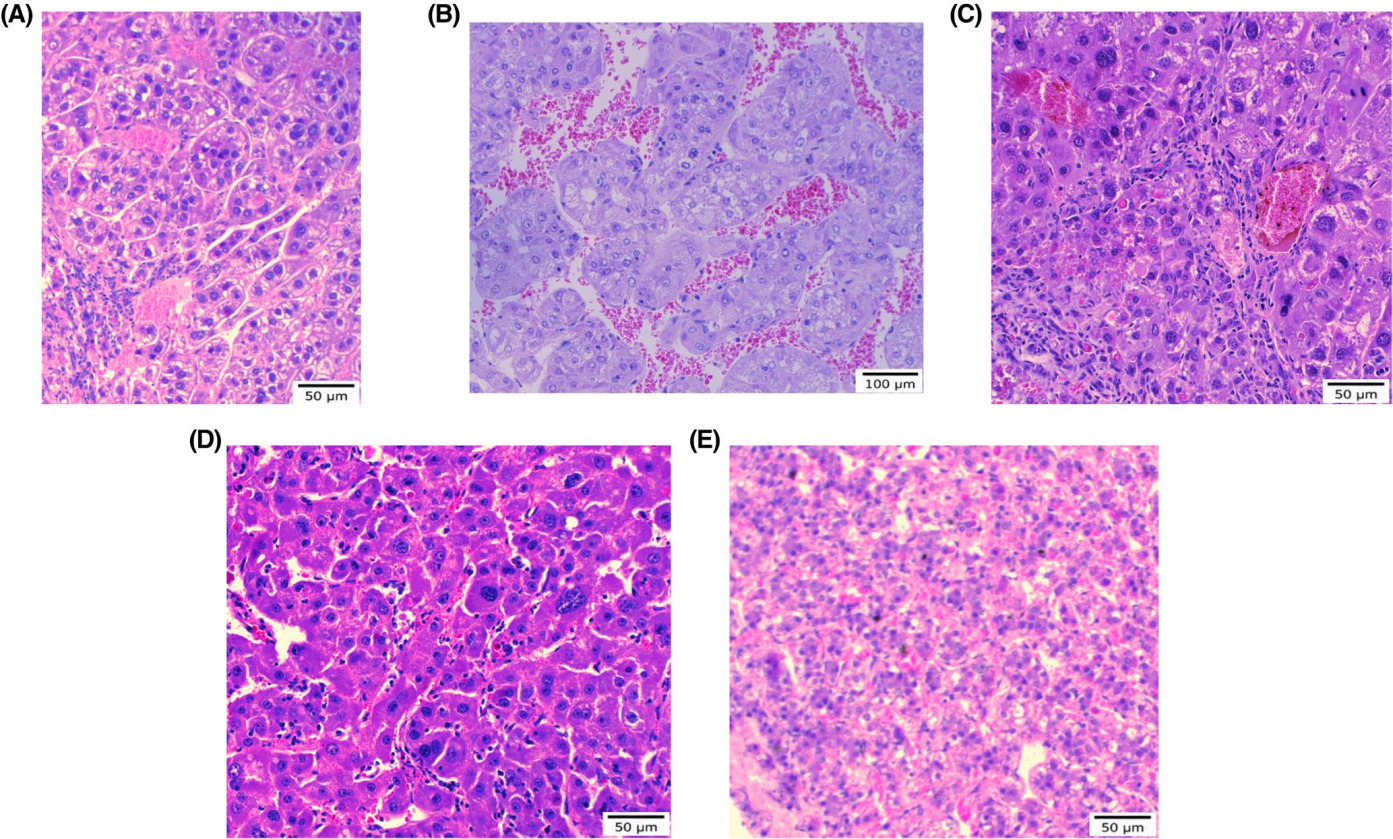
Representative hematoxylin and eosin (H&E)‐stained liver sections from tumor‐bearing *Smad4* ^+/−^ mice. Histopathological examination of liver sections confirmed hepatocellular carcinoma (HCC) in five cases. Shown are representative H&E‐stained sections from CC025 (A), CC010 (B), and CC005 (C) *Smad4* ^+/−^ mice, exhibiting trabecular architecture, nuclear atypia, and eosinophilic cytoplasm typical of HCC. (D) CC037. (E) Normal liver section from a tumor‐free littermate is shown for comparison.

Across the entire mouse cohort, spontaneous hepatic tumors were exclusively observed in the *Smad4* ^+/−^ genotype, with no tumors detected in any WT mice (0/239). Out of 260 *Smad4* ^+/−^ animals, eight of four different CC lines developed liver tumors, yielding a genotype‐wide incidence of 3.08%. Histological analysis confirmed HCC in five of these cases, whereas the remaining three exhibited gross hepatic lesions >3 mm with irregular morphology, consistent with previously established criteria for spontaneous liver neoplasia in mice. Representative histopathological findings from these cases are shown in Figures 2A‐E, demonstrating typical features of murine HCC, including trabecular architecture and nuclear atypia.

**FIGURE 3:**
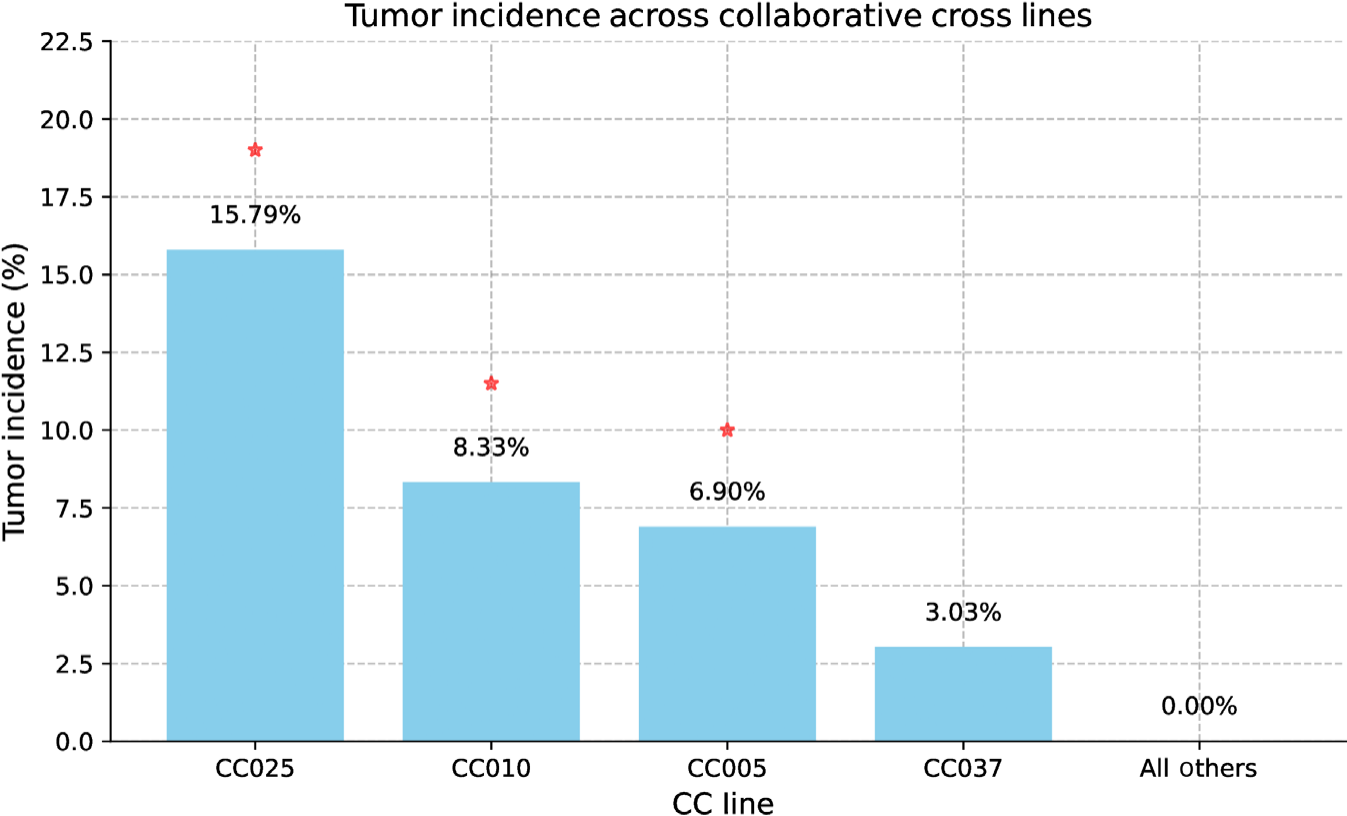
Tumor incidence across Collaborative Cross (CC) lines in *Smad4* ^+/−^ F1 mice. Bar plot illustrates the percentage of *Smad4* ^+/−^ mice with liver tumors across 14 CC lines. Tumors were observed in four lines: CC025, CC010, CC005, and CC037. Nominal enrichment was detected in CC025 (*p* = 0.0011), CC010 (*p* = 0.0173), and CC005 (*p* = 0.0241) relative to the pooled “All Other CC Lines” group. Raw *p*‐values are shown. After Bonferroni correction for 14 comparisons, only CC025 remained statistically significant (adjusted *p* = 0.0154). Error bars represent standard error.

When tumor incidence was stratified by CC line, we observed substantial variation, with tumors confined to four specific lines: CC025, CC010, CC005, and CC037. Notably, CC025 exhibited the highest incidence, with 3 of 19 *Smad4* ^+/−^ mice (15.79%) developing hepatic tumors. CC010 and CC005 showed elevated rates of 8.33% (2/24) and 6.90% (2/29), respectively, whereas CC037 had a single case among 33 *Smad4* ^+/−^ mice (3.03%). The remaining 10 lines showed no tumors in any *Smad4* ^+/−^ or WT mice. The distribution of liver tumor incidence across lines is illustrated in Figure 3, highlighting elevated susceptibility in CC025 and CC010 compared to the remaining lines.

To evaluate the statistical significance of these findings, Fisher's exact tests were applied comparing each tumor‐positive line against the pooled cohort of remaining *Smad4* ^+/−^ mice. CC025 (*p* = 0.0011), CC010 (*p* = 0.0173), and CC005 (*p* = 0.0241) showed nominal enrichment prior to correction for multiple testing, whereas CC037 was not statistically different (*p* = 0.1755). Following Bonferroni correction for 14 line‐specific comparisons, CC025 remained statistically significant (adjusted *p* = 0.0154), whereas CC010 (adjusted *p* = 0.2422), CC005 (adjusted *p* = 0.3374), and CC037 (adjusted *p* = 1.000) did not meet the corrected significance threshold. A detailed breakdown of tumor‐positive mice, incidence rates, and statistical significance across CC lines is presented in Table 1.

**TABLE 1:** Line‐specific incidence of liver tumors in Smad4^+/−^ F1 mice across Collaborative Cross (CC) lines.

CC Line	Total *Smad4* ^+/−^ mice	Tumor‐positive	Incidence (%)	Fisher's exact *p*‐value	Bonferroni‐adjusted *p*‐value
CC025	19	3	15.79	0.0011	0.0154
CC010	24	2	8.33	0.0173	0.2422
CC005	29	2	6.90	0.0241	0.3374
CC037	33	1	3.03	0.1755	1.000
All other CC lines	155	0	0.00	–	

*Note*: This table summarizes the number of tumor‐positive mice and incidence percentages for each Smad4^+/−^ F1 cohort derived from crosses between C57BL/6J‐Smad4^+/−^ males and individual CC lines. Only mice carrying the Smad4^+/−^ genotype were included. Tumor incidence (%) was calculated as the proportion of tumor‐positive mice out of the total Smad4^+/−^ mice per line. Statistical comparison for each line versus the pooled remainder of Smad4^+/−^ mice was performed using Fisher's exact test. Significance was determined at a threshold of 0.05. CC025 exhibited statistically significant enrichment for liver tumors after Bonferroni correction. CC010 and CC005 showed nominal enrichment but did not remain significant after correction for multiple testing. No tumors were observed in wild‐type (Smad4^+/+^) mice. The category ‘All Other CC Lines’ represents the combined data from the remaining CC lines included in the study (CC004, CC006, CC012, CC018, CC019, CC035, CC040, CC041, CC059, and CC084).

These findings are consistent with the presence of line‐specific genetic modifiers that may interact with heterozygous *Smad4* deletion to influence liver tumor susceptibility. Importantly, WT littermates from the same lines remained tumor free, reinforcing a gene‐by‐background interaction rather than a purely strain‐driven effect.

Furthermore, tumors were observed in both male and female mice within susceptible lines, suggesting that sex was not a major determinant in this context. One animal (mouse ID 228, CC025 background) exhibited HCC together with a large intestinal lesion identified at necropsy. Histopathological examination confirmed that the intestinal lesion was a nonmalignant polyp rather than a carcinoma. Because this observation was limited to a single animal, its biological significance remains uncertain.

These results suggest that heterozygous *Smad4* deletion alone is insufficient to induce HCC, and that additional genetic factors contribute to tumor susceptibility. The robust enrichment observed in CC025, together with the nominal enrichment observed in CC010 and CC005, identifies these lines as candidates for future systems genetics and modifier‐gene mapping studies.

## 4 DISCUSSION

This study demonstrates that partial loss of *Smad4*, a key mediator of TGF‐β signaling, can promote liver tumorigenesis in a host genetic background–dependent manner. Although heterozygous *Smad4* deletion alone is insufficient to drive HCC in a uniform genetic background, our findings from F1 crosses between *Smad4* ^+/−^ C57BL/6J mice and 14 diverse CC strains reveal strain‐specific susceptibility to spontaneous liver cancer. Notably, CC025 exhibited the highest tumor incidence (15.8%) and remained statistically significant after Bonferroni correction (adjusted *p* = 0.0154), whereas CC010 showed a higher incidence than the cohort average but did not remain significant after correction.

These findings suggest that the biological consequences of heterozygous *Smad4* deletion are highly dependent on genetic context, with host genetic background playing an important role in determining susceptibility to liver tumor development. *Smad4* acts as a tumor suppressor by mediating cytostatic and pro‐apoptotic effects of TGF‐β in hepatocytes, particularly during early stages of liver injury and regeneration.^33^, ^34^ In later stages, however, aberrant TGF‐β signaling may switch to a tumor‐promoting role via epithelial‐to‐mesenchymal transition, fibrosis, and immune modulation.^35^

Previous mouse models have established that complete loss of *Smad4* in hepatocytes, particularly in combination with oncogenic hits (e.g., *KRAS*, β‐catenin activation, or *Trp*53 loss), accelerates HCC formation.^36^, ^37^ In contrast, our study focuses on heterozygous loss, revealing that heterozygous *Smad4* knockout is not uniformly oncogenic but, instead, reveals hidden cancer susceptibility loci modulated by host genetics.

The CC panel used here represents >50 million segregating variants across eight founder strains, providing a powerful framework for identifying gene‐by‐background interactions.^11^, ^38^

Interestingly, no tumors were detected in WT littermates (0 of 239 mice), even in tumor‐prone lines, suggesting that heterozygous *Smad4* deletion is required for tumor formation in this model. This supports a gene–environment–genotype interaction, where *Smad4* loss unmasks strain‐dependent oncogenic potential.

Line CC025, in particular, warrants further investigation because it exhibited the highest tumor incidence among the evaluated CC lines. Future investigations incorporating transcriptomic, proteomic, or QTL mapping approaches may help identify the genetic factors and candidate modifier loci contributing to this increased susceptibility. This study complements our previous analysis of intestinal tumor susceptibility in the same F1 cohort, as described in the juvenile polyposis model (JPS) study.^14^ Notably, although intestinal polyps were widespread across CC lines in that study, hepatic tumors were rare and confined to only four lines (CC025, CC010, CC005, and CC037). This divergence underscores the tissue‐specific nature of genetic modifiers acting in concert with heterozygous *Smad4* deletion. For example, lines such as CC040 and CC059 exhibited high polyp burdens in the intestine but showed no liver tumors, whereas CC025, which ranked only eighth in intestinal polyp count, displayed the highest liver tumor incidence in this cohort. These results suggest that distinct, organ‐specific genetic pathways modulate tumor susceptibility. Although the limited number of tumor‐positive cases precluded robust quantitative estimation of heritability, the restriction of tumors to a small subset of CC lines supports an important role for host genetic background in modulating hepatic tumor susceptibility. In both tissues, incomplete penetrance was evident, highlighting the role of secondary stochastic or microenvironmental events beyond germline predisposition. Taken together, these findings suggest that although heterozygous *Smad4* deletion primes susceptibility, the manifestation of tumors is strongly shaped by tissue‐contextual genetic interactions. Consistent with this observation, one CC025 mouse developed HCC together with a large intestinal polyp, which was histologically confirmed to be nonmalignant.

Although CC lines are designed to be inbred, they typically retain 1%–3% residual heterozygosity. This partial genetic variation may contribute to phenotypic variability even among littermates, particularly for complex traits like tumor susceptibility. Thus, each CC line should be considered a genetically defined population rather than a fully isogenic clone.^39^

The finding that only a subset of *Smad4* ^+/−^ mice within susceptible lines developed liver tumors underscores the incomplete penetrance of the phenotype. This suggests that germline predisposition alone is insufficient, and tumorigenesis likely requires interaction with stochastic or microenvironmental factors—such as epigenetic shifts, immune dynamics, or somatic mutations.^40^, ^41^

Given the low number of tumor‐positive cases (8 of 499 mice), the study's power to detect modest genetic effects is limited. Although this constrains fine‐scale comparisons, the recurrent observation of tumors in specific lines across both sexes supports the biological significance of the findings.

Despite uniform housing and diet, subtle environmental influences such as cage microbiota, handling stress, or seasonal variation may affect phenotype expressivity. Although minor relative to genetics, such variables could impact tumor latency or penetrance.^42^, ^43^

Altogether, the identification of strain‐specific modifiers in a controlled genetic model strengthens the broader concept of gene–environment interplay in cancer. These results reflect patterns seen in humans, where inherited predisposition often requires additional somatic or environmental triggers to drive HCC.

A key limitation of this study is its retrospective design and the absence of histological confirmation for all tumor‐positive cases. Nonetheless, the observed gross liver lesions aligned with established morphological features of murine liver tumors, and representative histological analyses (H&E staining) confirmed malignancy in selected samples. Although the findings clearly demonstrate phenotype‐level interactions between *Smad4* genotype and genetic background, the study lacks molecular validation. Future investigations incorporating transcriptomic, proteomic, or QTL mapping approaches will be essential to identify underlying mechanisms and candidate genetic modifiers. An additional limitation of this study is that tumor latency could not be assessed. Because liver lesions were identified at necropsy, rather than through prospective monitoring, the timing of tumor onset and progression could not be determined accurately.

Previous studies have reported both tumor‐promoting and tumor‐suppressive functions of *Smad4* in HCC, highlighting the context‐dependent nature of TGF‐β/SMAD signaling. In experimental models, *Smad4* inhibition has been shown to suppress hepatocarcinogenesis. Our findings complement these observations by demonstrating that spontaneous liver tumor development in heterozygous *Smad4* knockout mice is strongly influenced by host genetic background. Although the present study was not designed to define the molecular mechanisms underlying these strain‐dependent differences, it identifies susceptible genetic contexts that may facilitate future investigations aimed at uncovering modifier loci and pathways involved in hepatocarcinogenesis.

## 5 CONCLUSION

Our results highlight the importance of host genetic context in shaping cancer susceptibility in the presence of heterozygous *Smad4* deletion. The finding that only specific CC lines developed tumors, despite identical breeding design and housing conditions, underscores the power of genetic diversity in modeling complex diseases. This study lays the groundwork for future identification of modifier loci, and ultimately, for understanding how genotype‐specific risk interacts with tumor suppressor pathways in hepatocarcinogenesis.

This work emphasizes the relevance of genetically diverse animal models, such as the CC, to capture human‐like heterogeneity in cancer risk and offers a compelling model for studying gene–environment interactions in liver cancer.

## AUTHOR CONTRIBUTIONS

**Aysar Nashef:** Conceptualization; investigation; writing – original draft; methodology. **Imad Abu‐Elnaaj:** Resources; funding acquisition. **Osayd Zohud:** Investigation; writing – original draft; methodology; data curation. **Fuad A. Iraqi:** Conceptualization; investigation; funding acquisition; writing – original draft; methodology; validation; visualization; software; writing – review and editing; project administration; supervision; resources.

## FUNDING INFORMATION

This study was supported by a core fund from Tel Aviv University and the Department of Oral and Maxillofacial Surgery, Baruch Padeh Medical Center, Poriya, Israel, and the Ministry of the Negev, Galilee, and National Resilience.

## CONFLICT OF INTEREST STATEMENT

Fuad A. Iraqi is an editorial board member of Animal Models and Experimental Medicine (*AMEM*) and a corresponding author of this article. To minimize bias, she was excluded from all editorial decision making related to the acceptance of this article for publication.

## ETHICS STATEMENT

All animal experiments conducted in this study adhered to national standards governing the care and utilization of laboratory animals. The experiment was thoroughly reviewed and received approval from Tel Aviv University's Institutional Animal Care and Use Committee (IACUC) under the designated reference number 01‐19‐044.

## Data Availability

The data that support the findings of this study are available on request from the corresponding author. The data are not publicly available due to privacy or ethical restrictions.
